# Association of plasma neurofilament light chain with disease activity in chronic inflammatory demyelinating polyradiculoneuropathy

**DOI:** 10.1111/ene.15496

**Published:** 2022-07-25

**Authors:** Mahima Kapoor, Aisling Carr, Martha Foiani, Amanda Heslegrave, Henrik Zetterberg, Andrea Malaspina, Laura Compton, Elspeth Hutton, Alexander Rossor, Mary M. Reilly, Michael P. Lunn

**Affiliations:** ^1^ Department of Neuromuscular Diseases University College London Queen Square Institute of Neurology London UK; ^2^ Department of Neurosciences Central Clinical School, Monash University, Alfred Centre Melbourne Victoria Australia; ^3^ Centre for Neuromuscular diseases National Hospital for Neurology and Neurosurgery London UK; ^4^ Department of Neurodegenerative Disease University College London Queen Square Institute of Neurology London UK; ^5^ UK Dementia Research Institute at University College London London UK; ^6^ Clinical Neurochemistry Laboratory Sahlgrenska University Hospital Mölndal Sweden; ^7^ Department of Psychiatry and Neurochemistry Institute of Neuroscience and Physiology, Sahlgrenska Academy at University of Gothenburg Mölndal Sweden; ^8^ Hong Kong Center for Neurodegenerative Diseases Hong Kong China; ^9^ University College London Queen Square Motor Neuron Disease Centre, Queen Square Institute of Neurology London UK; ^10^ Centre for Neuroscience and Trauma Blizard Institute, Barts and London School of Medicine and Dentistry, Queen Mary University of London London UK; ^11^ ALS Biomarkers Study University College London London UK; ^12^ Neuroimmunology and CSF Laboratory University College London Queen Square Institute of Neurology London UK

**Keywords:** CIDP, disease activity, IVIg, neurofilament light chain

## Abstract

**Background and purpose:**

This study was undertaken to explore associations between plasma neurofilament light chain (pNfL) concentration (pg/ml) and disease activity in patients with chronic inflammatory demyelinating polyradiculoneuropathy (CIDP) and examine the usefulness of pNfL concentrations in determining disease remission.

**Methods:**

We examined pNfL concentrations in treatment‐naïve CIDP patients (*n* = 10) before and after intravenous immunoglobulin (IVIg) induction treatment, in pNfL concentrations in patients on maintenance IVIg treatment who had stable (*n* = 15) versus unstable disease (*n* = 9), and in clinically stable IVIg‐treated patients (*n* = 10) in whom we suspended IVIg to determine disease activity and ongoing need for maintenance IVIg. pNfL concentrations in an age‐matched healthy control group were measured for comparison.

**Results:**

Among treatment‐naïve patients, pNfL concentration was higher in patients before IVIg treatment than healthy controls and subsequently reduced to be comparable to control group values after IVIg induction. Among CIDP patients on IVIg treatment, pNfL concentration was significantly higher in unstable patients than stable patients. A pNFL concentration > 16.6 pg/ml distinguished unstable treated CIDP from stable treated CIDP (sensitivity = 86.7%, specificity = 66.7%, area under receiver operating characteristic curve = 0.73). Among the treatment withdrawal group, there was a statistically significant correlation between pNfL concentration at time of IVIg withdrawal and the likelihood of relapse (*r* = 0.72, *p* < 0.05), suggesting an association of higher pNfL concentration with active disease.

**Conclusions:**

pNfL concentrations may be a sensitive, clinically useful biomarker in assessing subclinical disease activity.

## Graphical abstract

We explored the utility of plasma neurofilament light chain concentration (pNfL) in assessing disease activity in a cohort of patients with chronic inflammatory demyelinating polyradiculoneuropathy (CIDP) on maintenance intravenous immunoglobulin (IVIg). We demonstrated that pNfL concentration was significantly higher in unstable patients than stable patients. A pNFL concentration > 16.6 pg/ml identified unstable treated CIDP from stable treated CIDP. Also, there was statistically significant correlation between pNfL concentration at time of IVIg withdrawal and the likelihood of relapse (*r* = 0.72, *p* < 0.05), suggesting an association of higher pNfL concentration with active disease.

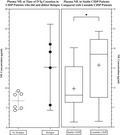

## INTRODUCTION

Chronic inflammatory demyelinating polyradiculoneuropathy (CIDP) is an acquired, demyelinating radiculoneuropathy commonly treated with immunomodulating drugs.^1^ Clinical outcome measurements remain the gold standard in assessing response to treatment and ongoing disease activity.^2^ However, these patient‐ and/or clinician‐reported tools have issues with sensitivity, specificity, and inter‐ and intraobserver variability. Blood biomarkers of direct neuronal damage might improve assessments of disease activity in CIDP, where differentiating patients in remission from those with active disease stabilized on maintenance therapy is a major challenge to clinicians and trial design.^3^

Neurofilaments, and specifically neurofilament light (NfL), have been shown to be useful biomarkers of axonal damage in central and peripheral nervous system disorders.^4^ No clinical biomarkers of Schwann cell damage currently exist, but axonal degeneration can be part of more florid demyelinating disease, and thus NfL might function as an indirect marker of disease activity.

The objective of this study was to explore the association between plasma neurofilament light chain (pNfL) concentration and disease activity in patients with CIDP and examine the potential usefulness of pNfL concentration as an available axonal biomarker in determining disease activity or remission.

## METHODS

### Patients and setting

In this prospective observational cohort study conducted between 2017 and 2019, we included CIDP patients who fulfilled the European Federation of Neurological Societies/Peripheral Nerve Society (EFNS/PNS) criteria managed by the neuromuscular unit at our centre.^5^ Patients were categorized into three cohorts:

- Treatment‐naïve patients: Newly diagnosed CIDP patients or recently relapsed patients untreated for >6 months, who were then treated with intravenous immunoglobulin (IVIg) alone (*n* = 10).
- CIDP patients on treatment: Patients on maintenance IVIg treatment only for >6 months. This group was divided into stable patients (*n* = 15; defined as clinical stability in subjective and objective clinical outcomes, no change in treatment regimen for >6 months) and unstable (*n* = 9; defined as a deterioration in subjective and objective clinical outcomes, requiring change in treatment regimen within the previous 6 months).
- Treatment withdrawal cohort: Clinically stable CIDP patients on maintenance IVIg only in whom we attempted to assess disease activity potentially suppressed by adequate treatment with a suspension of IVIg (*n* = 10). Relapse occurrence after treatment withdrawal was defined as any deterioration that required restarting treatment, as judged by the treating physician. The patients who did not relapse were deemed to be in remission and those who did were regarded as having active disease.

An equal number of age‐matched healthy controls (HC) were analysed for each subgroup. There was no overlap between groups. The study was approved by the local medical ethical committee (16/LO/1852), and written informed consent was obtained from all participants.

### Data Collection

Data were collected prospectively, including demographic features and disease and treatment variables. Disease variables included clinical phenotype, fulfilment of EFNS/PNS criteria, time since diagnosis, Medical Research Council sum score (MRC‐SS; out of 70, addition of power in bilateral first dorsal interossei to standard MRC‐SS) and disability scores (Inflammatory Rasch‐built Overall Disability Scale [I‐RODS]) at 3–6 months, routine clinical review, and/or at time of deterioration, and neurophysiology results (pre‐IVIg).^1^, ^5^, ^6^, ^7^

### Sample Collection

In treatment‐naïve patients, initial pNfL concentration was taken at diagnosis or prior to starting IVIg. Follow‐up pNfL concentration was taken on Day 1 of the third IVIg dose.

In treated patients, pNfL concentration was taken at routine clinical review (two to three times per year) for stable patients, and at clinical assessment when patients reported deterioration/fluctuation for unstable patients.

In the treatment withdrawal cohort, pNfL concentration was acquired at time of decision to suspend IVIg, made at a routine clinic appointment with the treating neurologist. The median frequency of IVIg in this group was every 6 weeks (range = 1–13 weeks). pNfL concentration was collected at a variety of time points within the maintenance IVIg treatment cycle, and patients had not missed a cycle of IVIg yet.

### Plasma NfL measurements

Samples were processed according to standard local protocols as described previously.^8^, ^9^ Plasma NfL concentration (pg/ml) was measured using the commercially available NF‐light kit on a Simoa HD‐1 Analyser, according to the manufacturer's instructions (Quanterix). The measurements were performed in one round of experiments using one batch of reagents by analysts who were blinded to clinical data. Intra‐assay coefficients of variation were <13%. The lower limit of quantification was 0.174 pg/ml.

### Statistical analysis

Descriptive statistics are shown as mean (±SD) or median (interquartile range) in continuous variables and as frequencies (percentages) in categorical variables. The comparison between quantitative variables was conducted by parametric (Student *t*‐tests) or nonparametric tests (Mann–Whitney *U*) as appropriate. Correlation between independent variables was explored with Spearman rho or Pearson correlation as appropriate. To identify the cutoff point with the best discriminative value of the continuous variable, receiver operating characteristic (ROC) curve analysis was carried out. A value of *p* ≤ 0.05 was considered significant. Statistical analysis was performed using SPSS version 27 (IBM, 2020) and GraphPad Prism version 9.2.0.

## RESULTS

### Treatment‐naïve patients

Patient characteristics and basic demographics of the treatment‐naïve group are shown in Table 1.

**TABLE 1:** Baseline characteristics of patients with CIDP

Characteristic	Untreated CIDP, *n* = 9	Stable CIDP patients on IVIg treatment, *n* = 15	Unstable CIDP patients on IVIg treatment, *n* = 9	Successful IVIg cessation patients [remission], *n* = 6	CIDP patients with active disease, *n* = 4
Age of patients, years, mean (SD)	57.9 (11.6)	58.6 (13.6)	59.6 (15.4)	47.1 (13.6)	59.8 (12.3)
Gender, F/M	1/8	4/11	3/6	3/3	2/2
Age of healthy controls, years, mean (SD)	56.4 (12.3)	58.1 (13.7)			
pNfL, pg/ml, mean (SD)	17.5 (5.7)	9.5 (6.1)	17.0 (7.9)	6.8 (2.2)	15 (6.9)
Years from diagnosis, median (IQR)	0.06 (0.35)	6.5 (5.9)	4.2 (3.2)	6.16 (6.52)	8.56 (3.59)
Onset of symptoms to plasma sampling, months, median (IQR)	28.7 (59.3)				
Phenotype: typical, *n* (%)	5 (55.6%)	14 (93.3)	8 (88.9)	5 (83.3)	3 (75)
Phenotype: atypical, *n* (%)	4 (44.4%)	1 (6.7)	1 (11.1)	1 (16.7)	1 (25.0)
2010 EFNS/PNS criteria: definite CIDP, *n* (%)	5 (55.6%)	9 (60.0)	7 (77.8)	5 (83.3)	2 (50)
2010 EFNS/PNS criteria: possible CIDP [0 = probable CIDP], *n* (%)	4 (44.4%)	6 (40.0)	2 (22.2)	1 (16.7)	2 (50)
I‐RODS, mean (SD)	39.4 (6.9)	69.3 (18.4)	52.0 (12.6)		
MRC‐SS [max = 70], mean (SD)	67.9 (1.5)	66.8 (3.3)	63.7 (6.5)		
IVIg ratio, g/kg/month, median (IQR)		1.38 (0.81)	1.40 (1.34)		
Time to relapse, days, median (IQR)					171.50 (219.25)

*Note*: Treatment‐naïve patients: newly diagnosed CIDP patients or recently relapsed patients untreated for >6 months. CIDP patients on treatment: patients on maintenance IVIg treatment only for >6 months. Stable patients: defined as clinical stability in subjective and objective clinical outcomes, no change in treatment regimen for >6 months. Unstable patients: defined as a deterioration in subjective and objective clinical outcomes, requiring change in treatment regimen within the previous 6 months. Treatment withdrawal cohort: clinically stable CIDP patients on maintenance IVIg only in whom we attempted to assess disease activity potentially suppressed by adequate treatment with a suspension of IVIg. Relapse: defined as any deterioration that required restarting treatment, as judged by the treating physician. Remission: those who did not relapse.

Abbreviations: CIDP, chronic inflammatory demyelinating polyradiculoneuropathy; EFNS/PNS, European Federation of Neurological Societies/Peripheral Nerve Society; F, female; IQR, interquartile range; I‐RODS, Inflammatory Rasch‐built Overall Disability Scale; IVIg, intravenous immunoglobulin; M, male; MRC‐SS, Medical Research Council sum score; pNfL, plasma neurofilament light chain.

**FIGURE 1:**
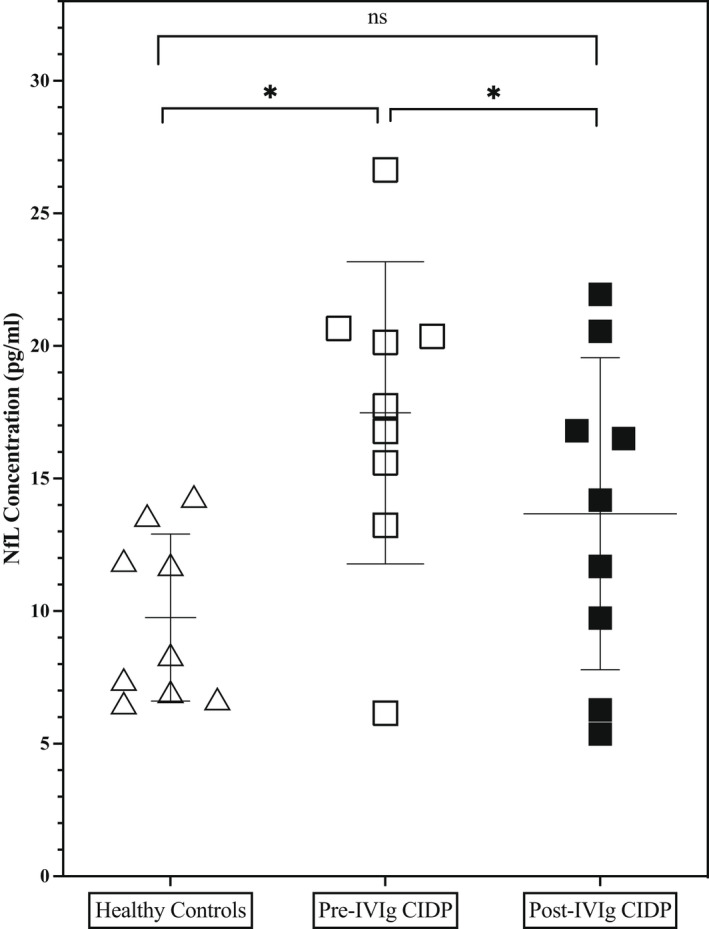
Plasma neurofilament light (NfL) in controls, before and after intravenous immunoglobulin (IVIg) induction in chronic inflammatory demyelinating polyradiculoneuropathy (CIDP) patients. ns, not significant. *Statistically significant

**FIGURE 2:**
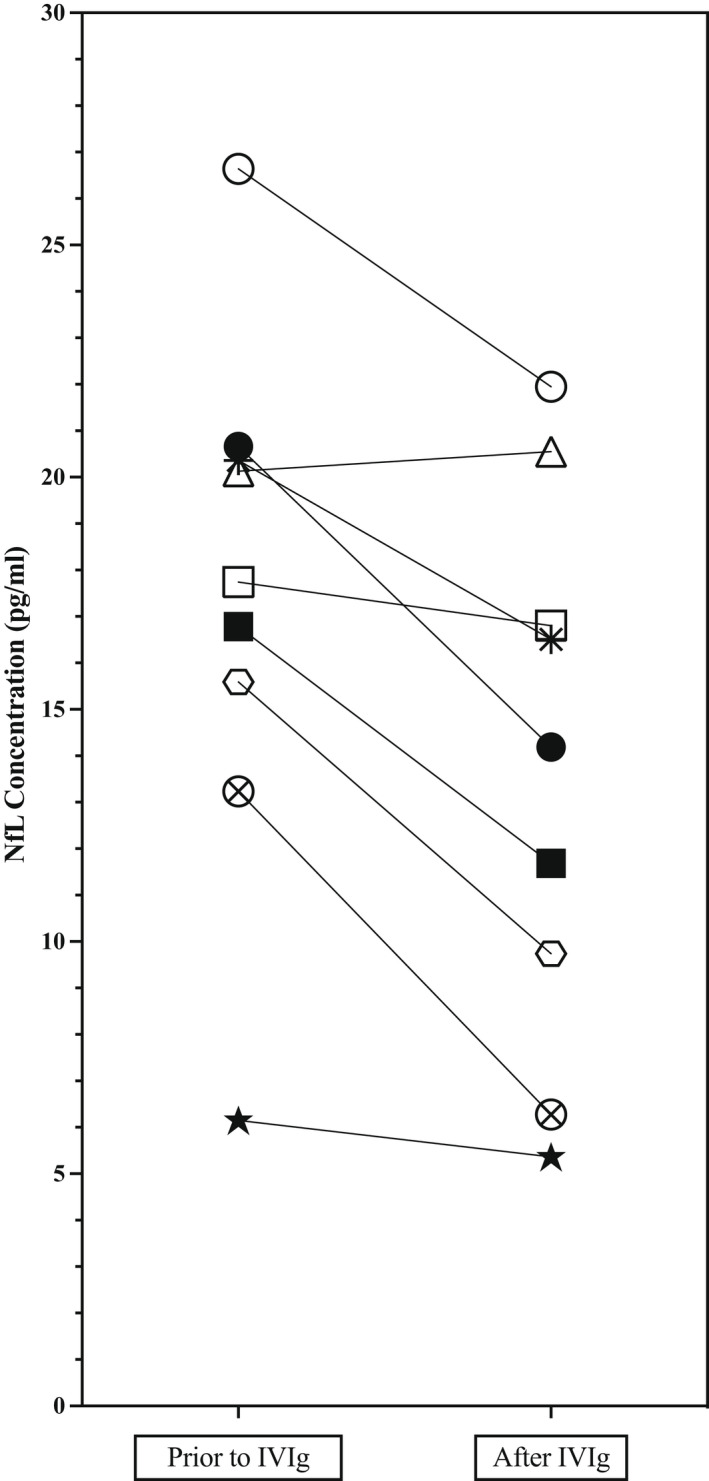
Plasma neurofilament light (NfL) in chronic inflammatory demyelinating polyradiculoneuropathy patients before and after intravenous immunoglobulin (IVIg) induction

A Mann–Whitney *U* test showed that pNfL concentration (pg/ml) was higher in treatment‐naïve CIDP patients (*n* = 9, mean = 17.5 [11.6] than HC (*n* = 9, mean 9.8 [3.1], *p* < 0.01; Figure 1). After IVIg induction, the NfL concentration fell to levels more comparable with HC values (mean = 13.7 [5.9] vs. 9.8 [3.1], *p* = 0.1; Figures 1 and 2). Wilcoxon matched‐pairs signed rank test demonstrated that pre‐IVIg pNfL concentration was significantly higher than post‐IVIg pNfL concentration in these patients (*n* = 9, mean = 13.7 [5.7] vs. 13.7 [5.9], *p* < 0.05).

**FIGURE 3:**
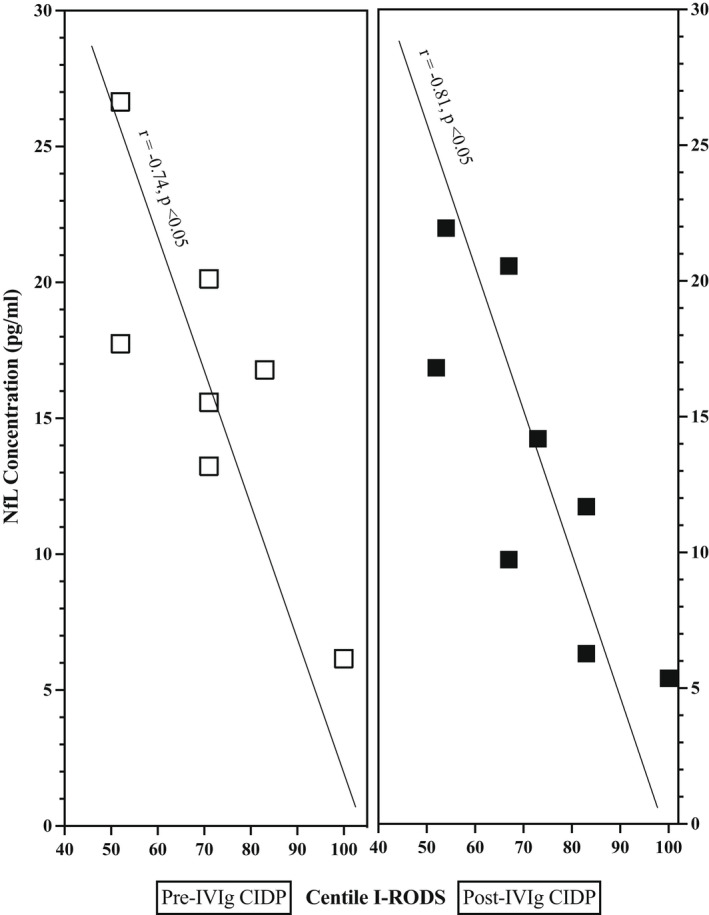
Plasma neurofilament light (NfL) concentration in relation to Inflammatory Rasch‐built Overall Disability Scale (I‐RODS) before and after intravenous immunoglobulin (IVIg) induction. CIDP, chronic inflammatory demyelinating polyradiculoneuropathy

Higher pretreatment pNfL concentration correlated with lower pretreatment I‐RODS (*r* = −0.74, *p* < 0.05), suggesting more disability with more ongoing damage. A similar negative correlation was seen posttreatment with lower pNfL concentration and higher I‐RODS (*r* = −0.81, *p* < 0.05; Figure 3), also suggesting clinical validity of the measurement.

There was no correlation between pretreatment pNfL concentration and pretreatment neurophysiological measures of axonal damage (mean proximal or mean distal summated compound muscle action potential [CMAP] in upper and lower limbs, data not shown). Posttreatment pNfL concentrations and posttreatment neurophysiological measures of axonal damage were not analysed due to incomplete data and inconsistent times between treatment and neurophysiology.

### Treated patients: Clinically stable and unstable

**FIGURE 4:**
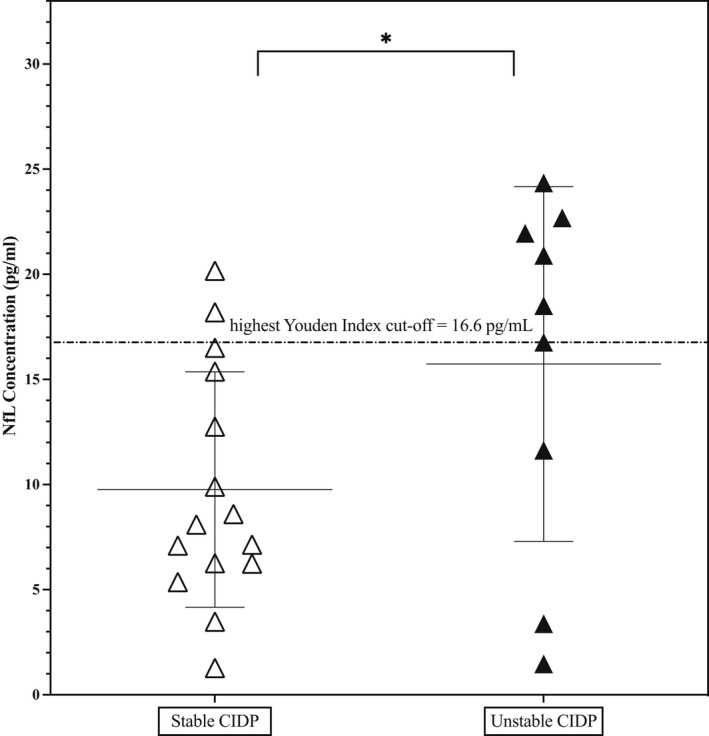
Plasma neurofilament light (NfL) in stable chronic inflammatory demyelinating polyradiculoneuropathy (CIDP) patients compared with unstable CIDP patients (both groups on maintenance intravenous immunoglobulin). *Statistically significant

Some baseline patient and clinical disease characteristics differed in the treated stable and unstable patients according to the definitions above (Table 1). The ages of patients and proportion of patients with typical CIDP phenotype and IVIg ratio were similar in both groups. However, stable patients had longer duration of disease and were less impaired and less disabled than those with unstable disease. For example, I‐RODS was significantly lower (worse) in the unstable group (*p* < 0.05). pNfL (pg/ml) was higher in patients predesignated as unstable (*n* = 9) than stable patients (*n* = 15; mean = 15.7 [8.4] vs. 9.8 [5.6], *p* < 0.05; Figure 4). There was no groupwise difference between mean pNfL concentration in either group of stable or unstable treated patients compared to age‐matched HC. This suggests some protection from axonal damage with treatment even in the clinically unstable individuals but also potentially highlights issues of small group size.

A pNfL concentration value > 16.6 pg/ml identified unstable treated CIDP from stable treated CIDP (sensitivity = 86.7%, specificity = 66.7%, area under the ROC curve = 0.73). However, the confounding role of differences in clinical outcome measures (MRC‐SS and I‐RODS) between these groups in pNfL concentrations is unknown (ie, whether pNfL concentration depends on disease stability and clinical outcome measurements, and which is more influential).

**FIGURE 5:**
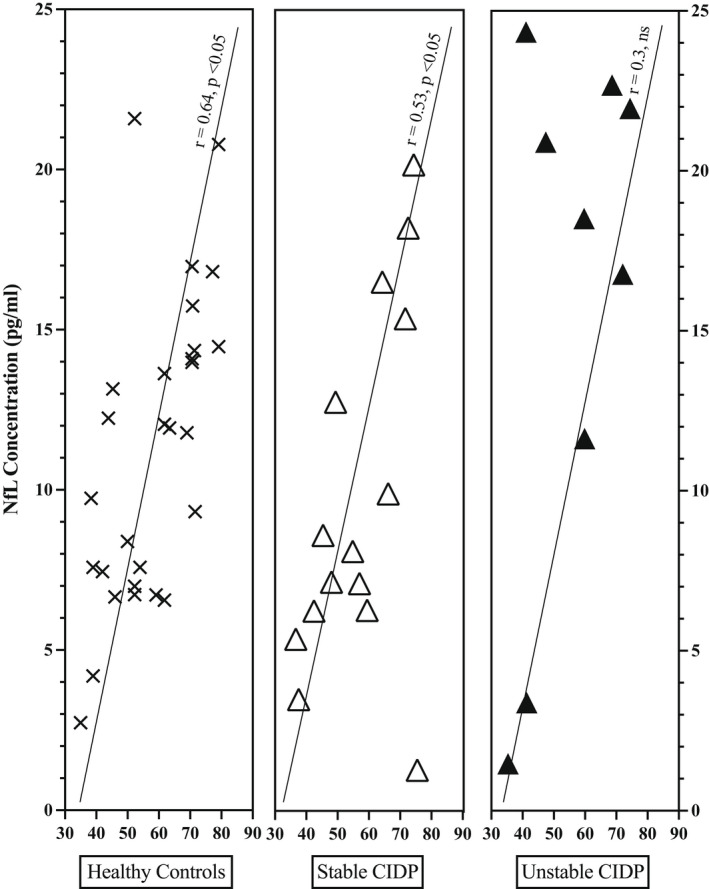
Plasma neurofilament light (NfL) concentration in relation to age of controls and stable and unstable chronic inflammatory demyelinating polyradiculoneuropathy (CIDP) patients. ns, not significant

The previously documented correlation between age and pNfL concentration in HC was also demonstrated in this study (*r* = 0.64, *p* < 0.001).^10^ There was also a correlation between age and pNfL concentration in the stable treated CIDP (*r* = 0.53, *p* < 0.05) but not in unstable treated CIDP group (Figure 5).

### Determining disease activity in treated CIDP by treatment withdrawal

**FIGURE 6:**
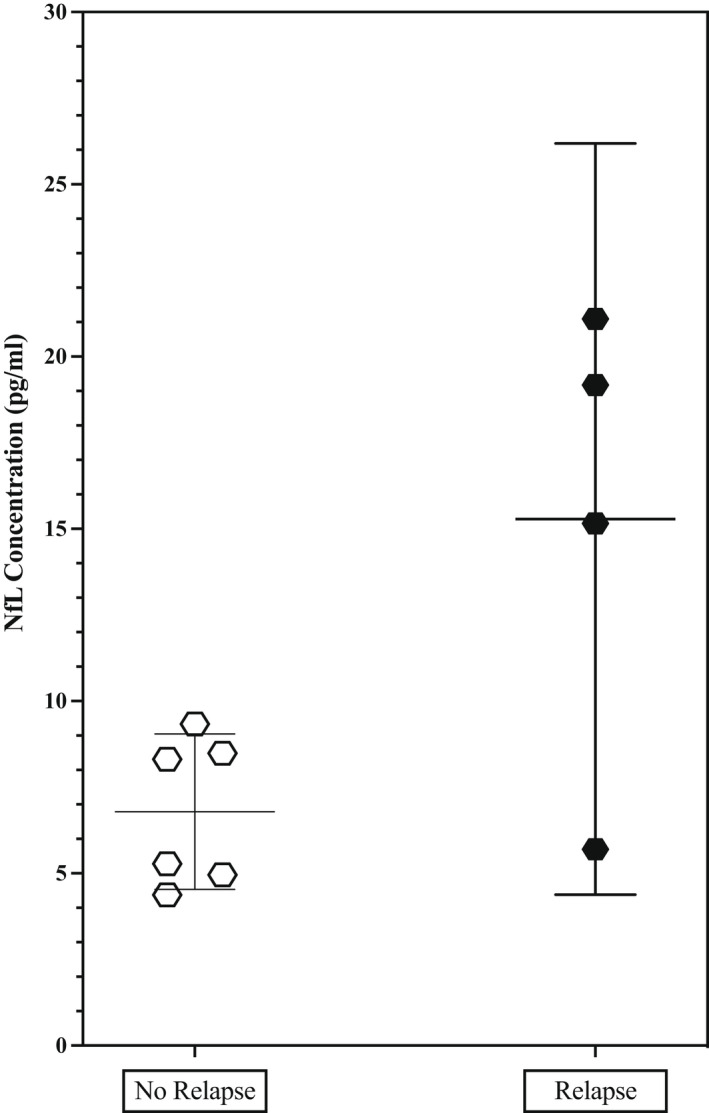
Plasma neurofilament light (NfL) at time of intravenous immunoglobulin cessation in chronic inflammatory demyelinating polyradiculoneuropathy patients who did and did not relapse

Clinical decline on treatment withdrawal or suspension is the current approach to differentiating CIDP patients with active disease requiring ongoing maintenance treatment from those in remission.^11^, ^12^ Using this approach on 10 stable, treated CIDP patients, we identified four with active disease and six in remission. There were no demographic, disease, or treatment differences between those subsequently found to have active disease and those in remission once IVIg was withdrawn. Those who failed treatment withdrawal were, on average, 10 years older and had an average of 3 years shorter disease than those who were in disease remission. The mean pNfL concentrations at time of decision to withdraw IVIg (no IVIg missed yet) in the whole group was 10.2 pg/ml (6.1). A one‐way analysis of covariance was conducted to compare the difference in pNfL concentrations between the group in remission and the group of those who had relapsed while controlling for age. There was no statistically significant difference between the pNfL concentrations at time of decision to withdraw IVIg (*p* = 0.11; Figure 6). However, there was a statistically significant correlation between relapse after treatment withdrawal and baseline pNfL concentrations (*r* = 0.72, *p* < 0.05). Therefore, although the absolute pNfL concentration did not differ between those who did and did not relapse, there was an association of higher pNfL concentration with active disease.

## DISCUSSION

In this study, we show that pNfL concentrations are higher in treatment‐naïve CIDP patients than in controls and settle to levels similar to HC after treatment. During maintenance treatment with IVIg, a cutoff of >16.6 pg/ml indicates a patient is likely to have unstable or undertreated disease. Higher pNFL concentrations prior to treatment withdrawal correlate with the likelihood of relapse, suggesting pNfL concentration as a potentially useful biomarker of disease activity and response to treatment in CIDP.

A strong correlation with I‐RODS, a widely used disability score, demonstrates clinical validity. This study also suggests pNfL concentration is a more sensitive indicator of neuronal damage than CMAPs. The sensitivity of the Simoa allows for differentiation between stable and unstable individuals within a cohort of treated patients and shows promise in the prediction of remission in a clinically stable, treated group. The identification of these subclinical differences in individuals in whom maintenance treatment can mask disease activity has important implications for therapeutic decision‐making and clinical trial design. Using this biomarker can potentially avoid functional deterioration caused by treatment cessation in those with active disease, save on Ig spending, and reduce potential for Ig‐related adverse events in those in remission.

NfL concentrations in different publications cannot be directly compared, as we calculated plasma concentrations and other studies used serum samples. Mean pNfL concentrations were found to be 10% lower than in serum in previous studies examining the analytical performance of the assay.^13^ Even with this “conversion” taken into consideration, our pNfL concentrations in treatment‐naïve patients, treated patients, and those patients in remission were lower than expected compared to other studies.^10^, ^14^, ^15^ One study that included 29 CIDP patients starting treatment, 24 patients on maintenance IVIg treatment, and 27 patients in remission also found significantly greater serum NfL concentration in the untreated group compared to HC, and no difference in concentrations between the treated or previously treated groups and HC. Patients with active disease also had higher NfL concentrations compared to those with stable disease, and the majority of patients who had induction treatment and clinically responded had normal NfL concentrations at follow‐up.^16^ They also compared the groups' concentrations to age‐specific reference values from their laboratory. Validation of this by other groups, and on a larger scale, will be useful to determine sensitivity/specificity of blood NfL in diagnosis of CIDP.

We found a difference in pNfL concentrations between untreated and stable CIDP patients, which two other research groups did not.^16^, ^17^ The Netherlands' patients were similar to ours in terms of age of patients, delay from symptoms onset to blood sampling in the untreated group, and duration of CIDP in the maintenance group. Their groups are larger than ours (*n* = 29 for untreated patients vs. *n* = 10 in ours, and *n* = 24 for maintenance patients vs. *n* = 15 in ours), and potentially our small sample size and using plasma samples has contributed to this difference.

Other groups have also correlated NfL concentrations with electrophysiological findings and biopsy features of demyelination and/or axonal degeneration with varying results.^10^, ^17^

Godelaine et al. approached using serum NfL to prognosticate on disease activity and treatment response differently from our method. They collected baseline serum samples on 76 CIDP patients, most of whom were already on treatment, with a median disease duration of 6 years and repeat samples 1 year later. Patients who had a decrease of at least 4 points on an 80‐point MRC‐SS at repeat sampling were deemed as having disease progression, and if there had been a change in treatment modality (IVIg, plasma exchange, other) within the year, they were deemed as nonresponders. Baseline serum NfL concentrations were significantly increased in patients with disease progression compared with those without disease progression.^15^ Again, this suggests that blood NfL can measure subclinical disease activity. Their definition of treatment response potentially reflects different clinical practices; they do not mention whether a change in dose (especially for IVIg) was attempted before changing treatment modality.

In our study, the suggestion that pNfL concentration is higher in those who are clinically stable, but have active disease as proven by a relapse upon withdrawal of treatment, compared to those who do not relapse, suggests that subclinical demyelination is likely resulting in active secondary axonal degeneration. This process appears to continue even while patients are on treatment and being monitored regularly. There are many mechanisms by which demyelination is thought to result in axon damage, such as by increasing energy demand, mitochondrial dysfunction, and loss of metabolic and trophic support for the axon.^18^ Identifying axonal loss is essential, as it is a key marker of prognosis in patients with CIDP.^19^, ^20^, ^21^, ^22^, ^23^, ^24^ The suggestion of ongoing axonal degeneration in clinically stable patients has not been consistently proven by other studies that investigated neurophysiological changes in CIDP patients on treatment. One study of 60 patients on long‐term maintenance immunosuppressive or immunomodulatory treatment with an average of 4–5 years of follow‐up found that those who clinically improved on treatment had a trend toward improvement in upper and lower limb motor CMAPs, and sensory nerve action potentials between first neurophysiology and last neurophysiology.^25^ In another study of 11 CIDP patients treated with IVIg for at least 1 year, there was a suggestion of increase in distal CMAPs between pretreatment neurophysiology and at last follow‐up.^26^ Our findings may be novel because of the sensitivity of the Simoa in measuring NfL concentrations, and the clinical consequence of our findings are unknown (ie, the impact of pNfL concentration on prognosis and response to treatment, and the influence of the difference in pre‐ and posttreatment pNfL concentration on prognosis).

We acknowledge that pNfL concentration is not specific to disease activity in CIDP or a direct measure of myelin‐directed damage. It is associated with age and potentially influenced by other unknown factors, as reflected by the broad range of concentrations measured in this study, even when patients were categorized according to clinical and therapeutic status. In a cohort of individuals without neurological conditions, a nonlinear association between pNfL concentration and age was reported.^27^ We saw correlation between age and pNfL concentration in the treated and clinically stable patients but not within the clinically unstable subgroup. In the treatment‐naïve group, those with typical CIDP according to 2010 EFNS/PNS diagnostic criteria demonstrated the largest magnitude of change in pNfL concentrations between pre‐ and posttreatment measures.^5^ The multifactorial influences on pNfL concentrations highlight potential limitations to the utility of pNfL concentration in a heterogenous disorder such as CIDP. Also, time to diagnosis and treatment, pattern of disability, response to treatment, treatment regimens, and tolerance can all vary, which may impact pNfL concentrations.

This is a small study, where statistically significant differences between groups may not have been demonstrated due to limited numbers of patients.

As our understanding of NfL improves, we will be able to decide whether it is a useful biomarker. In CIDP, if its role in subclinical disease can be confirmed, it would be clinically very useful, as other tests such as neurophysiology are unreliable predictors of clinical disease activity. If our cutoff for NfL in those at risk of clinical destabilization can be confirmed, our ability to provide responsive and proactive care would be greatly improved. Further work is required to explore the optimal manner of integration of this blood test into clinical decision‐making. Nevertheless, this work provides intriguing preliminary data to suggest pNFL concentration as a sensitive, clinically informative biomarker in CIDP.

## AUTHOR CONTRIBUTIONS

**Mahima Kapoor:** Conceptualization (supporting); formal analysis (lead); investigation (supporting); methodology (supporting); writing – original draft (lead); writing – review and editing (lead). **Aisling Carr:** Conceptualization (supporting); methodology (supporting); resources (supporting); supervision (supporting); writing – review and editing (supporting). **Martha Foiani:** Data curation (equal); formal analysis (equal); investigation (equal); project administration (equal); validation (equal). **Amanda Heslegrave:** Data curation (equal); formal analysis (equal); investigation (equal); methodology (equal); project administration (equal); resources (equal). **Henrik Zetterberg:** Formal analysis (equal); funding acquisition (equal); investigation (equal); methodology (equal); project administration (equal); resources (equal); validation (equal). **Andrea Malaspina:** Resources (equal). **Laura Compton:** Project administration (equal); resources (equal). **Elspeth Hutton:** Methodology (equal); project administration (equal); supervision (supporting). **Alexander Rossor:** Conceptualization (equal); formal analysis (equal); investigation (equal); methodology (equal); project administration (equal); validation (equal); writing – review and editing (equal). **Mary M Reilly:** Conceptualization (equal); funding acquisition (equal); project administration (equal); resources (equal); supervision (equal); validation (equal); writing – review and editing (equal). **Michael P Lunn:** Conceptualization (equal); data curation (equal); formal analysis (equal); investigation (equal); methodology (equal); project administration (equal); resources (equal); supervision (supporting); validation (equal); writing – review and editing (equal).

## CONFLICT OF INTEREST

M.K. reports Grifols sponsorship for meeting attendance. A.C. reports Grifols sponsorship for meeting attendance and honoraria from CSL and Lupin for an advisory role. M.P.L. was a Primary Investigator in studies for CSL Behring, UCB Pharma, Novartis, Octapharma. He has also received ad hoc consulting fees from CSL Behring and UCB and an honorarium from Terumo BCT. H.Z. reports, outside the submitted work, institutional research support and support to attend scientific meetings from Bayer Healthcare, with honoraria for lectures from Bayer Healthcare and consultancy fees from UCB Biopharma paid to University College London Hospitals Charity. H.Z. has served on scientific advisory boards and/or as a consultant for Abbvie, Alector, Annexon, Artery Therapeutics, AZTherapies, CogRx, Denali, Eisai, Nervgen, Novo Nordisk, Pinteon Therapeutics, Red Abbey Labs, Passage Bio, Roche, Samumed, Siemens Healthineers, Triplet Therapeutics, and Wave, has given lectures in symposia sponsored by Cellectricon, Fujirebio, Alzecure, Biogen, and Roche, and is a cofounder of Brain Biomarker Solutions in Gothenburg, which is a part of the GU Ventures Incubator Program (outside submitted work). The remaining authors declare that the research was conducted in the absence of any commercial or financial relationships that could be construed as a potential conflict of interest.

## Data Availability

Participants of this study did not agree for their data to be shared publicly, so supporting data is not available.
